# Amikacin Dosing and Monitoring in Spinal Cord Injury Patients: Variation in Clinical Practice Between Spinal Injury Units and Differences in Experts' Recommendations

**DOI:** 10.1100/tsw.2006.44

**Published:** 2006-02-17

**Authors:** Subramanian Vaidyanathan, Charles Peloquin, Jean-Jacques Wyndaele, Andrew Z. Buczynski, Yaniv Almog, Sophia L. Markantonis, Vidya Jayawardena, Bakul M. Soni, Joan Cannon, Joan Vidal

**Affiliations:** ^1^ Regional Spinal Injuries Centre, District General Hospital, Southport, Merseyside PR8 6PN, UK; ^2^ Pharmacokinetics Laboratory, Rm. K-424a, National Jewish Medical and Research Center, 1400 Jackson Street, Denver, CO 80206, USA; ^3^ Centrum Urologische Revalidatie, Universitair Zieckenhuis Antwerpen, 10 Wilrijkstraat, B 2650 Edegem, Belgium; ^4^ Metropolitan Rehabilitation Centre, Department of Neuro-Urology, Konstancin, Poland; ^5^ Medical Intensive Care Unit, Soroka University Medical Center, P.O.B. 151, Beer-Sheva 84101, Israel; ^6^ Laboratory of Biopharmaceutics and Pharmacokinetics, School of Pharmacy, University of Athens, Panepistimiopolis 15771, Athens, Greece; ^7^ VA McGuire Medical Center, 1201 Broad Rock Blvd, Richmond, VA 23113, USA; ^8^ Pharmacy Service, Edward Hines VA Hospital, Hines, IL 60141, USA; ^9^ Neurorehabilitation Hospital Institut Guttmann, Barcelona, Spain

**Keywords:** spinal cord injury, amikacin

## Abstract

The objective of this article was to determine the current practice on amikacin dosing and monitoring in spinal cord injury patients from spinal cord physicians and experts. Physicians from spinal units and clinical pharmacologists were asked to provide protocol for dosing and monitoring of amikacin therapy in spinal cord injury patients. In a spinal unit in Poland, amikacin is administered usually 0.5 g twice daily. A once-daily regimen of amikacin is never used and amikacin concentrations are not determined. In Belgium, Southport (U.K.), Spain, and the VA McGuire Medical Center (Richmond, Virginia), amikacin is given once daily. Whereas peak and trough concentrations are determined in Belgium, only trough concentration is measured in Southport. In both these spinal units, modification of the dose is not routinely done with a nomogram. In Spain and the VA McGuire Medical Center, monitoring of serum amikacin concentration is not done unless a patient has renal impairment. In contrast, the dose/interval of amikacin is adjusted according to pharmacokinetic parameters at the Edward Hines VA Hospital (Hines, Illinois), where amikacin is administered q24h or q48h, depending on creatinine clearance. Spinal cord physicians from Denmark, Germany, and the Kessler Institute for Rehabilitation (West Orange, New Jersey) state that they do not use amikacin in spinal injury patients. An expert from Canada does not recommend determining serum concentrations of amikacin, but emphasizes the value of monitoring ototoxicity and nephrotoxicity. Experts from New Zealand recommend amikacin in conventional *twice*- or *thrice-daily* dosing because of the theoretical increased risk of neuromuscular blockade and apnea with larger daily doses in spinal cord injury patients. On the contrary, experts from Greece, Israel, and the U.S. recommend once-daily dosing and determining amikacin pharmacokinetic parameters for each patient. As there is considerable variation in clinical practice across spinal units and experts differ on ideal dosing and monitoring of amikacin therapy in spinal cord injury patients, there is an urgent need to develop best-practice guidelines.

### INTRODUCTION

Injury to the cervical and upper dorsal spinal cord produces a variety of changes in the physiological function of different body systems. Patients with spinal cord injury, both acute and long standing, comprise a therapeutically unique and distinct population[1]. A significant increase in the volume of distribution of drugs such as the aminoglycosides (gentamicin, amikacin, and tobramycin), which are highly distributed into the extracellular fluid space and minimally biotransformed, may be anticipated in patients with chronic spinal cord injury[2]. Therefore, amikacin dosing regimens, developed in able-bodied humans, may demonstrate diminished efficacy when extrapolated uncritically in spinal cord injury patients[3] or spinal cord injury patients may develop a high serum concentration of aminoglycoside[4].

Amikacin is used in the treatment of serious infections caused by gentamicin-resistant Gram-negative bacilli in spinal cord injury patients. We wished to find out the degree of consensus in the use of amikacin in spinal units across Europe and the U.S. Therefore, we asked spinal cord physicians and experts their current practice and recommendations.

### METHODS

Spinal cord physicians in Europe and the U.S. who have published articles on urinary infection were asked to provide details of the use of amikacin in spinal cord injury patients and justify their clinical practice in their respective spinal injury centers. Physicians and clinical pharmacologists who have published articles on dosing/monitoring of aminoglycosides were asked whether they would use amikacin in spinal cord injury patients and, if so, what should be the protocol for dosing and monitoring of amikacin therapy in spinal cord injury patients.

### RESULTS

Clinical practice relating to administration of amikacin to spinal cord injury patients in spinal units located in Belgium, Poland, Spain, the U.K., and the U.S. is summarized in Table 1. Recommendations made by experts from Canada, Greece, Israel, New Zealand, and the U.S. on the use of amikacin in spinal cord injury patients are summarized in Table 2. Full details are available in the Appendix.

### DISCUSSION

This study shows that there is considerable variation in clinical practice across spinal units and even experts differ on ideal dosing and monitoring of amikacin therapy in spinal cord injury patients. It is important to collect information prospectively on (1) clinical and microbiological efficacy of amikacin and (2) ototoxicity and nephrotoxicity, especially when amikacin is used without the monitoring of serum

**TABLE 1**
**Summary of Clinical Practice Relating to Administration of Amikacin to Spinal Cord Injury Patients in Different Spinal Injury Centers of Europe and the U.S.**

| Geographic Location of Spinal Injury Center | Is Amikacin Used in Spinal Cord Injury Patients? | How is Amikacin Administered; Once Daily or in Divided Doses? | Serum Samples for Amikacin Concentrations; Target Peak and Trough Concentrations | How is Amikacin Dose Modified? | Are Pharmacokinetic Parameters Calculated for Each Patient or is a Nomogram Used? |
|---|---|---|---|---|---|
| Edegem, Belgium | Yes | Once daily, 1.5 g/day | Peak concentrations are measured daily; target: 15–30 mg/l; trough concentration is determined after 48 h; target: equal to or less than 2.5 mg/l. | If there is worsening of kidney function as revealed by declining creatinine clearance, the interval between two doses is increased or amikacin is stopped. | Pharmacokinetic parameters are not calculated for each patient; a nomogram is not used. |
| Konstancin, Poland | Yes | 0.5 g twice daily | No facility for measuring amikacin concentration. | — | Pharmacokinetic parameters are not calculated for each patient; a nomogram is not used. |
| Southport, U.K. | Yes | 1 g once daily | Only trough concentrations are determined; target: <5 mg/l. | If trough concentrations are high, amikacin is discontinued. | Pharmacokinetic parameters are not calculated for each patient; a nomogram is not used. |
| Barcelona, Spain | Yes | Once daily | Monitoring is done only in patients with renal insufficiency. | — | Pharmacokinetic parameters are not calculated for each patient; a nomogram is not used. |
| Richmond, Virginia | Amikacin is used only if there is no other choice. | Once daily; dose depends on the creatinine clearance. | Monitoring is done only in patients with renal insufficiency. | — | Pharmacokinetic parameters are not calculated for each patient; a nomogram is not used. |
| Hines, Illinois | Yes | The interval between doses depends on creatinine clearance. | Trough concentration is determined before the third dose and peak concentration, 1 h after infusion of third dose; target peak: 20–40 mg/l; troughs: <2.5 mg/l | Modification is done using pharmacokinetic parameters (Vd, ke, t1/2). | Nomogram of Edward Hines VA Hospital is used. |
| Herne, Germany | No | | | | |
| Copenhagen, Denmark | No | | | | |
| West Orange, New Jersey | No | | | | |

**TABLE 2**
**Summary of Recommendations of Experts from Canada, Greece, Israel, New Zealand, and the U.S. Regarding the Use of Amikacin in Spinal Cord Injury Patients**

| Name of Expert and Country of Residence | Is Once-Daily Amikacin Regimen Recommended in Spinal Cord Injury Patients? | How Should Serum Samples be Taken for Determining Amikacin Concentration? | What Should be Done to Monitor Amikacin Therapy? | Calculation of Amikacin Pharmacokinetic Parameters |
|---|---|---|---|---|
| Prof. James McCormack, Canada | Yes | It is not necessary to determine serum concentrations of amikacin. | Serum creatinine should be measured every 2–3 days. Doses of amikacin should be adjusted on the basis of changes in renal function. Clinical assessment for ototoxicity is vital. | Not recommended. |
| Sophia Markantonis, Greece | Yes | Serum samples are taken postinfusion, 1, 3, 6, and 10 h after the onset of infusion and just before the next dose. | Nephrotoxicity should be monitored by measuring serum creatinine, urine output, and creatinine clearance at least every second day during therapy and after discontinuation of amikacin. | Amikacin pharmacokinetic parameters for each patient are calculated by using a one-compartment model with the WinNonlin software program. The individual parameters are used to revise initial dose (when necessary) to achieve a target peak concentration of 45–60 mg/l. |
| Yaniv Almog, Israel | Yes | One hour after initiation of infusion, first serum sample is taken to measure peak concentration; another sample is taken between 6–18 h to determine trough concentration. Initial dose: 15 mg/kg once daily. Target peak concentration: 60 mg/l. | Amikacin dose is adjusted according to peak concentration and interval between doses is adjusted according to trough concentration. If trough concentration is >1 mg/l at 24 h, the subsequent dose is delayed to 36 or even 48 h. It is not unusual to give 2 g of amikacin every 36 h. | Volume of distribution and elimination half-life is calculated. |
| Profs. Barclay and Begg, New Zealand | No. Conventional twice or three times daily dosing is recommended. | Blood samples are taken at 0.5 h after the dose and at the trough, just before the next dose. | Monitor amikacin concentrations every 2–3 days, or daily if unstable renal function, aiming for peaks and troughs of 20–30 and 1–8 mg/l, respectively. Monitor serum creatinine every 2–3 days. | Doses of amikacin and/or dose interval should be adjusted using Bayesian forecasting, or the Sawchuk-Zaske's method of log-linear regression assuming a one-compartment model. |
| Charles Peloquin, U.S. | Yes | After the first dose, serum samples are taken 2 and 6 h postinfusion to avoid the distribution phase. | Monitor BUN, creatinine, K+, Ca++, and Mg++ about weekly. Baseline and biweekly audiogram for long courses. Vestibular testing on physical exam as often as practical. | Pharmacokinetic calculations are made (WinNonlin or USC*PACK MAP Bayesian). The targeted back-calculated end of infusion Cmax is 35–45 mg/l for daily doses. Target Cmax at least 12 times the MIC. |

concentrations and dosage is not adjusted according to individual pharmacokinetic parameters. At some centers, amikacin is prescribed infrequently, i.e., only in clinical situations when no other antimicrobial is effective. Therefore, it may not be possible to conduct a prospective study at a single spinal injuries center. We propose an international electronic database to record prospectively the details of amikacin therapy in spinal cord injury patients. The proposed database may include the following: (1) details of amikacin dosing, including serum amikacin concentrations and pharmacokinetic parameters for individual patients, when available; (2) clinical and microbiological efficacy; (3) monitoring for ototoxicity and nephrotoxicity; and (4) adverse events directly attributable to amikacin therapy.

Data collected over approximately 12 months and analyzed should provide valuable information that could substantiate the case for individual pharmacokinetic monitoring of amikacin in spinal cord injury patients. On the contrary, such monitoring for each patient may not be considered essential if standardized amikacin therapy results in satisfactory clinical and microbiological outcomes.

### ACKNOWLEDGMENTS

The first author is grateful to Associate Professor Murray Barclay and Professor Evan Begg, Department of Clinical Pharmacology, Christchurch Hospital, Christchurch, New Zealand, and Professor James P. McCormack, Faculty of Pharmaceutical Sciences, University of British Columbia, Vancouver, BC, Canada for their valuable comments.

### APPENDIX I

### Clinical Practice — Centrum Urologische Revalidatie, Universitair Zieckenhuis Antwerpen, Edegem, Belgium

Amikacin is prescribed to spinal cord injury patients in whom urinary tract infection is resistant to more frequently used antibiotics, and antimicrobial sensitivity has shown that the uropathogen is sensitive to amikacin and treatment is considered necessary because of symptomatic infection. Once-daily regimen is preferred as its efficacy is equal to multiple dosing and literature showed reduced nephrotoxicity[5]. Amikacin is administered usually at 1.5 g/day or at a dosage depending on serum creatinine concentration. If serum creatinine is normal, amikacin is given 1.5 g once daily for 5 days intravenously. Trough concentration is determined after 48 h; peak concentration is measured daily at 30 min after administration.

The creatinine clearance is calculated as 140 – age in years × weight in kilo divided by 72 × serum creatinine (mg/100 ml). In women, multiplying the value by 0.85 makes correction.

Trough concentration is considered good if the concentration is lower or equal to 2.5 mg/l. Target peak concentration is 15–30 mg/l. If an exact body weight is not available, body weight is estimated by clinical examination, e.g., medium, small, or large build. Daily dose of amikacin is limited to a maximum of 2 g in a person with a large body build. If the trough concentration is double the permitted concentration, the dose is halved. If the peak concentration is too low, the dose of amikacin is increased to a maximum of 2 g once daily.

Modification of the dose is not routinely done with a nomogram. If there is any worsening kidney function as revealed by declining creatinine clearance, the interval between the two doses is increased or the treatment is stopped altogether.

### Clinical Practice — Department of Neuro-Urology, Metropolitan Rehabilitation Centre, Konstancin, Poland

Aminoglycosides are used only for selected patients who have urinary infection with bacteria resistant to other conventional antibiotics. In the last 30 years, two young tetraplegic men became deaf after 2 weeks of treatment with gentamicin 80 mg twice daily in the Metropolitan Rehabilitation Centre. Because of that, use of gentamicin for spinal cord injury patients was stopped in 1991. Some spinal cord injury patients, after multiple treatments of their urinary infections with many different antibiotics, develop urine infection with bacteria resistant to most antibiotics. Therefore, when there is no other choice, amikacin is prescribed if the organism is sensitive to amikacin. In this situation, amikacin is administered usually 0.5 g twice daily; a once-daily regimen of amikacin (1.0 g once daily) is never used. Amikacin is prescribed only for patients with normal serum creatinine concentration. Amikacin is contraindicated in spinal cord injury patients with impairment of kidney function. One gram of amikacin, once daily, is never used in spinal cord injured patients. Amikacin is always prescribed in divided doses: 0.5 g every 8 or 12 h, which is considered safer. There are already many risk factors for upper urinary tract deterioration in spinal cord injury people; therefore, these patients should not be subjected to another risk factor viz. amikacin-induced nephrotoxicity. Further, there is no facility for measuring concentration of antibiotics in serum. Therefore, estimation of amikacin concentration is not carried out.

### Clinical Practice — Northwest Regional Spinal Injuries Centre, Southport, U.K.

The use of amikacin is restricted to clinical situations when the uropathogen is not sensitive to any other antimicrobial. Usually spinal cord injury patients are prescribed 1 g of amikacin once daily, administered as an infusion over a period of 20–30 min. A serum sample is taken to measure trough concentration of amikacin, but subsequent doses are not modified using a nomogram or any other formula. Desired trough concentration is less than 5 mg/l. If amikacin concentrations are high, the drug is discontinued.

### Clinical Practice — Spinal Cord Unit, Neurorehabilitation Hospital Institut Guttmann, Barcelona, Spain

Amikacin is used in spinal cord injury patients. Once-daily dosing is practiced; amikacin is never administered in divided doses. Monitoring of amikacin concentration is carried out only in special situations, for example, patients with renal insufficiency or pediatric cases. The majority of patients have normal renal function and dose adjustments based on amikacin concentrations are not required in these patients. An important aspect of clinical practice is that amikacin is avoided in those spinal cord injury patients who have received botulinum toxin (for management of spasticity or detrusor hyperreflexia) within the last 3 weeks.

### Clinical Practice — VA McGuire Medical Center, Richmond, Virginia

At the VA McGuire Medical Center, Richmond, Virginia, the Infectious Disease Service does not generally recommend amikacin. The VA Medical Center formulary does not contain amikacin, whereas gentamicin is included in the formulary. Amikacin is used only if there is no other choice. Because of potential nephrotoxicity of amikacin, the use of amikacin is restricted to conditions such as urinary infection by gentamicin-resistant Gram-negative bacilli when it is not possible to prescribe other antibiotics such as ceftriaxone because of the patient’s allergy to cephalosporins. The dose of amikacin depends on creatinine clearance. Generally, amikacin is given once daily. Serum samples for measuring peak and trough concentrations of amikacin are not taken unless a patient has renal impairment. The dose of amikacin is modified if required, depending on the severity of infection and clinical condition.

### Clinical Practice — Edward Hines VA Hospital, Hines, Illinois

Amikacin is used in spinal cord injury patients at the Hines VA hospital. "Once-daily" dosing is practiced, but the nomenclature is sometimes inappropriate as some patients are prescribed q48h dosing due to poor renal function. The aminoglycoside dosing nomogram of the Edward Hines VA Hospital is followed along with monitoring protocol. Essentially, the initial amikacin dose is based on a milligram per kilogram basis. The interval is determined by using estimated creatinine clearance (CrCl) utilizing the Cockroft-Gault equation[6] or a 24-h measured creatinine clearance, if available. It is important to note that estimated CrCl in spinal cord injury patients has been found to be misleading[7,8]; therefore, it is important to monitor amikacin concentrations. Amikacin is administered q24h (for CrCl: >60 ml/min), q36 (for CrCl: 40–59 ml/min), or q48h (for CrCl: <40 ml/min). The q36h dosing is not favored, as q36h dosing leaves room for error. Therefore, if the CrCl falls in the 40 to 59 ml/min range, the dose is typically increased slightly and the q48h interval is utilized. Once a patient is initiated on an amikacin, amikacin concentrations (trough and peak) are measured around the third dose (if the patient is receiving it q24h) or around the second dose (if receiving q48h). The rationale for obtaining concentrations around the second dose (if the patient is receiving the q48h interval) is to determine if the patient is receiving sub- or supratherapeutic doses on day 4 of therapy, rather than day 6 of therapy. Serum samples for trough concentrations are obtained immediately before the third (or second) dose, and peak concentration 1 h after the infusion of the third (or second) dose. When the amikacin concentrations are known, the dose/interval is adjusted, if necessary, according to pharmacokinetic parameters (Vd, ke, t1/2); please refer to Appendix II for details on the calculations of these parameters.

All patients in the hospital (regardless of being a spinal cord injury patient or not) are prescribed amikacin according to the aminoglycoside nomogram and are monitored through the Pharmacy Pharmacokinetic Service. Amikacin concentrations are measured around the second or third dose (as described above). Target amikacin concentrations are peaks of 20–40 mg/l and troughs <2.5 mg/l. If the dose and/or interval are adjusted based on amikacin concentrations, further serum samples are taken to measure peak and trough concentrations around the second or third dose. Once therapeutic concentrations of amikacin are reached, serum concentrations are checked once weekly. The patient's serum creatinine is monitored while on amikacin therapy; if there is a significant change in the serum creatinine, serum samples are taken to check peak and trough concentrations of amikacin.

### Clinical Practice — Denmark, Germany, and Kessler Institute for Rehabilitation, New Jersey

Physicians from the Urologische Klinik der Ruhr-Universitat Bochum, Marienhospital, Herne, Deutschland (Dr. Jürgen Pannek); the Clinic for Spinal Cord Injuries, The NeuroScience Centre, Rigshospitalet, Copenhagen University Hospital, Copenhagen, Denmark (Dr. Fin Biering-Sørensen); and the Kessler Institute for Rehabilitation, West Orange, New Jersey (Dr. Todd Linsenmeyer) stated that they do *not* use amikacin in spinal cord injury patients.

### Expert Opinion — James McCormack, B.Sc. (Pharm), Pharm.D., Professor, Faculty of Pharmaceutical Sciences, University of British Columbia, Vancouver, BC, Canada

There is little evidence surrounding monitoring of amikacin; therefore, the best one can do is to extrapolate data from other aminoglycosides, e.g., gentamicin[9]. Serum creatinine should be measured every 2–3 days; if the serum creatinine increases 25% or so over baseline, the physician should be alert for further increases; if serum creatinine goes up by 50%, the need for amikacin therapy should be reassessed and strong consideration should be given to using an alternative agent. Doses of amikacin should be adjusted on the basis of changes in renal function and the need for the drug. The best dosage adjustment is to decrease the dose to zero as soon as possible. The patient should be asked about tinnitus, loss of hearing, a sense of fullness in the ears, headache, nausea and vomiting, light-headedness, vertigo, and ataxia. Since there are no studies that look at different ways of monitoring for ototoxicity, it is just common sense to ask the patient about signs and symptoms of a potential toxicity, as would be done for any other drug. Asking patients for symptoms of ototoxicity does not really cost anything; it keeps everyone thinking about the potential adverse effect and it is something that can be done on a daily basis. The problem with weekly auditory testing is that it can easily miss the problem if, say, ototoxicity starts on a Tuesday and the testing is done every Monday. A recent meta-analysis (64 studies, 7586 patients) showed that in patients with sepsis, the addition of an aminoglycoside to beta-lactam monotherapy did not produce any difference in mortality; in fact, clinical failure was higher in the group who received the combination of aminoglycoside and beta-lactam antibiotic[10]. The authors of this recent publication state: "in the treatment of sepsis, the addition of an aminoglycoside to beta-lactam therapy should be discouraged." This is an important finding; what it means at a minimum is that continued long-term treatment of amikacin should not be acceptable, especially given that ototoxicity typically occurs during long-term therapy. If one is monitoring for efficacy and toxicity on a daily basis and attempting to get the patient off the aminoglycoside as soon as possible, there is little if any reason or evidence to support doing concentrations[9,11].

### Recommendations — Dr. Sophia Markantonis, PhD Assistant Professor, Laboratory of Biopharmaceutics and Pharmacokinetics, School of Pharmacy, University of Athens, Panepistimiopolis, Athens, Greece

The disposition of aminoglycoside antibiotics in patients with spinal cord injury is different from that of able-bodied individuals. Therefore, dosage regimens for spinal cord injury patients should be individualized based on age, anthropometric indices, renal function, and level of spinal cord injury. Population pharmacokinetic models provide mean parameter estimates linked to informative covariates such as those mentioned above, e.g., age, gender, disease states, etc. Thus, a properly developed population pharmacokinetic model would be useful for the selection of dosages of amikacin in patients with spinal cord injury. Faced with a lack of population models, we necessarily turn to standard regimens of once-daily dosing. Amikacin once-daily dosage regimens, which have been used in research protocols, range between 11–30 mg/kg/day. The 15-mg/kg/day dose is the most widely used, while doses over 20 mg/kg/day are administered to patients with neutropenia and other immunodeficiencies.

Amikacin regimens should be adjusted to maintain peak concentrations of 60 ± 15 mg/l. Based on our previous study[12], I would begin with a dose between 12–15 mg/kg/day once daily, depending on renal function, which is determined by measurement of creatinine clearance from 24-h urine collection, not from serum creatinine. Patients with creatinine clearance <60 ml/min should not follow this protocol, but one based on dosage regimens for patients with renal insufficiency.

Plasma amikacin steady-state concentrations are determined on the second day by taking serum samples postinfusion, 1, 3, 6, and 10 h after the onset of infusion and just before the next dose. Sampling times may vary with the exception of the post- and preinfusion samples. Amikacin pharmacokinetic parameters for each patient are calculated by using a one-compartment model with the WinNonlin (version 3.0, Pharsight Corporation, CA) software program. The individual parameters are used subsequently to revise initial doses (when necessary) to achieve a target true peak concentration between 45–60 mg/l depending, once again, on renal function.

Also, if minimum inhibitory concentrations (MIC) of the microorganisms are available and Cmax/MIC values can be calculated, the initial dosage of amikacin should be adjusted to achieve a Cmax/MIC ratio of >8:1 to 10:1. Of course, monitoring of clinical and microbiological response to amikacin therapy is essential.

Nephrotoxicity should be monitored by measuring serum electrolytes, urea, creatinine and urine output or, ideally, creatinine clearance (calculated from 24-h urine collections) at least every second day during therapy, and after discontinuation of amikacin. In our previous study[12], we had patients with multiple injuries including spinal cord injuries, but these subjects were critically ill patients with multiple problems (severe infections, hemodynamic disturbances, changing body weight, etc.) including multiple drug therapy. Therefore, the subjects of our previous study differ from spinal cord patients with no serious co-morbidity who are prescribed amikacin for urinary infection. We have not dealt with such patients.

### Recommendations — Dr. Yaniv Almog, Director, Medical Intensive Care Unit, Soroka University Medical Center, Beer-Sheva, Israel

There are no data or clinical trials that specifically address dosing and monitoring of amikacin in spinal cord injury patients. Therefore, it is pertinent to refer to a prospective controlled trial of 81 patients with suspected or documented Gram-negative infections who were treated with either gentamicin or amikacin[13]. In the study group (pharmacokinetic dosing) of 43 patients, plasma aminoglycoside concentrations were determined 1 h after initiation of drug infusion and 8 to 16 h later to estimate the elimination half-life and volume of distribution from which the subsequent dosage schedule was calculated. Target peak plasma concentrations were 20 mg/l for gentamicin and 60 mg/l for amikacin. Target trough concentrations were <1 mg/l for both drugs. The control group (fixed once-daily dosing) consisted of 38 patients who were prescribed single daily doses of gentamicin or amikacin. Although the pharmacokinetic group received significantly greater doses of aminoglycosides than did the once-daily group, the incidence of nephrotoxicity (> or = 25% increase in serum creatinine concentration or a serum creatinine concentration > or = 1.4 mg/dl) was significantly lower in the pharmacokinetic group (5% [2/43] vs. 21% [8/38], $p$ = 0.03). These results suggest that individualized pharmacokinetic dosing of aminoglycosides reduces the incidence of nephrotoxicity and allows the use of greater doses of aminoglycosides.

The following recommendations are based on personal experience and practice as well as the reported literature related to use of amikacin in critically ill medical and surgical patients. There is no reason to believe that spinal cord injury patients should be treated in any different way in this respect.

Initial dose should be 15 mg/kg of amikacin, which practically translates to 1 g once daily for an average patient. Amikacin is administered over a period of 30 min. Half an hour later, i.e., 1 h after initiation of infusion, the first serum sample is taken to measured peak concentration of amikacin. Anywhere between 6 to 18 h later, another serum sample is taken to determine trough concentration of amikacin. Target peak concentration is 60 mg/l Volume of distribution and elimination half-life are calculated using a simple formula. Practically, the dose is adjusted according to the peak concentrations and the interval between doses is adjusted according to the trough concentration. For example, if 1 g of amikacin was given and the peak concentration was 30 mg/l, then the dose is doubled (attempting to reach a peak concentration of 60 mg/l). Adjustment for the interval between doses is trickier as the trough concentration should be less than 1 mg/l at 24 h. If the trough concentration is higher than1 mg/l at 24 h, the subsequent dose is delayed to 36 or even 48 h. It is not unusual for us to give 2 g of amikacin every 36 h.

The above protocol has been followed for once-daily amikacin dosing in the Soroka University Medical Center for many years. With the above amikacin once-daily regimen in very sick patients, many of whom have received vasopressor concomitantly, nephrotoxicity was not encountered in any patient. Therefore, the above-mentioned regimen for amikacin once-daily dosing is a very safe and practical mode of administering amikacin.

### Expert Opinion — Associate Professor M.L. Barclay and Professor E.J. Begg, Department of Clinical Pharmacology, Christchurch Hospital, Christchurch, New Zealand

For all aminoglycosides, including amikacin, conventional two or three times daily dosing is recommended because of the theoretical increased risk of neuromuscular blockade and apnea with larger daily doses in spinal injury patients. The possibility of apnea with amikacin is extrapolated from the clinical experience with other aminoglycosides. Doses of amikacin can be adjusted using Bayesian forecasting or the Sawchuk-Zaske's method of log-linear regression assuming a one-compartment model. Monitoring should use conventional target peak and trough concentrations. If the spinal cord clinician decides to use once-daily dosing, then the amikacin AUC (area under the amikacin serum concentration curve) should be monitored, with a target of 150–200 mg/l/h for the 24 h AUC for amikacin.

$$\text{Second dose} = \{\text{target AUC/measured AUC}\} \times \text{first dose}$$

Calculation of a patient's 24-h AUC is given in Appendix 1 of Begg et al.[14]. This method requires measurement of two drug concentrations: one approximately 0.5 h after the end of the infusion and another at a later time (6–22 h) depending on renal function.

### Recommendations — Charles Peloquin, Pharm.D, Director, Infectious Disease, Pharmacokinetics Laboratory, National Jewish Medical and Research Center, Denver, Colorado

The initial dose for the once-daily regimen in spinal cord injury patients should be the same as for other patients (15 mg/kg of amikacin often is used)[15]. After the first dose, serum samples are taken 2 and 6 h postinfusion to avoid the distribution phase[16] and to capture half-life. If sampling is performed immediately at the end of a 60-min infusion, distributional concentrations in sera are obtained and such data cannot be handled with standard one-compartment approaches. For large, once-daily doses of amikacin, at least 90 min should elapse prior to collecting the first serum sample. Early collection of serum may lead to falsely high Cmax/MIC ratio estimates[16].

Using amikacin concentrations at 2 and 6 h postinfusion, pharmacokinetic calculations can be made using either linear regression or Bayesian approaches. For daily amikacin doses of 15 mg/kg, the targeted back-calculated end of infusion Cmax is 35–45 mg/l, as described in our recent publications (note that amikacin and streptomycin have identical pharmacokinetic profiles)[17,18].

Dose modification for amikacin depends on the goals of treatment and how aggressively the physician wishes to treat the infection[19]. In general, amikacin is used with a beta-lactam, so it is not necessary to have the amikacin present all of the time. Hence, once-daily dosing of amikacin with Cmax about 12 times the MIC should be effective for most types of infections.

Although it is a reasonable approach to follow the Hartford Hospital once-daily aminoglycoside nomogram and change the time interval between two doses, if indicated, I find it preferable to do calculations using the USC*PACK software from the University of Southern California, which is available at http://www.lapk.org. USC*PACK is very good for the Bayesian approach and WinNonlin http://www.pharsight.com/ is very good for the log-linear and nonlinear regression approach.

Until results of more clinical trials are available, the best practice for the present would be to follow the method described above, which is very flexible. With serum samplings taken after the first dose, adjustments can be made very early in treatment. Recommendations on use of aminoglycosides are described in two recent chapters[20,21].

## APPENDIX II

### Aminoglycoside dosing in Edward Hines VA Hospital, Hines, IL 60141, USA

#### *High-Dose/Once-Daily Aminoglycoside Dosing*

*Rationale*

Achieve a high peak/Minimum Inhibitory Concentration ratio with a near zero trough concentrations

Optimize the concentration-dependent kill pharmacodynamics

Minimize risk of toxicity

| Drug | Desired Peak (mg/L) | Desired Trough (mg/L) |
|---|---|---|
| Gentamicin/Tobramicin | 10-15 | <1.0 |
| Amikacin | 35-40 | <2.5 |

Not Indicated:

**Synergy (e.g. endocarditis, Enterococcal bacteremia)**
***For endocarditis dose 1 mg/kg q8h or q12h (depending on renal function);***
***Aim for a peak of 3-5 and a trough of <1.***

*Dosing*

Initial Dosing

**Step 1: calculate creatinine clearance (CrCl) (ml/min) by Cockroft Gault equation**

$$\frac{(140 - \text{age}) \times \text{Ideal Body Weight}}{\text{Serum Creatinine} \times 72} \quad \textit{(x 0.85 for females)}$$

**Step 2: Calculate the patient's dosing weight**

For dosing, use ideal body weight in kg unless the patient is obese or weighs less then their IBW
For obese patients (patients ≥ 130% of IBW), use the "adjusted" body weight calculation:
IBW + [0.4 x (Actual BW-IBW)]=adjusted body weight
For patient's who weigh less than their IBW, use their actual body weight

**Step 3: Calculate the dosage based on the appropriate body weight and then determine the dosing interval based on the CrCl**

| CrCl ml/min | >60 | 40-59 | 20-39 |
|---|---|---|---|
| Gentamicin | q24h | Q36h | q48h |
| Amikacin | q24h | Q36h | q48h |

| | Dose Range | UTI | Bacteremia | Pneumonia |
|---|---|---|---|---|
| Amikacin | 7.5-15 mg/kg | 7.5 mg/kg | 10 mg/kg | 15 mg/kg |
| Gentamicin | 3-5 mg/kg | 3 mg/kg | 4 mg/kg | 5 mg/kg |
| Tobramycin | 3-5 mg/kg | 3 mg/kg | 4 mg/kg | 5 mg/kg |

**Adjusting a Dose Based on Levels**

**Drawing Levels**
**Draw a trough right before 2nd or 3rd dose**
**Draw a peak 1 hour after infusion of 2nd or 3rd dose**
**30 minute infusion for gentamicin and tobramycin**
**1 hour infusion for amikacin**

**Step 1: Vd (L) = Total Dose/ (Peak-Trough)**

**Step 2:** $k_e$ (hours$^{-1}$) $= \dfrac{\ln(\text{peak/trough})}{T^*}$

***(T= $\tau$ - t') where $\tau$ = dosing interval in hours, and t' = infusion period in hours.**

**Step 3:** $t_{1/2}$ **(hours)=** $0.693/k_e$

**Step 4:** ***New*** **Dose (mg) = Desired Peak x Vd**

**Step 5:** ***New*** **Interval (hours) =** $t_{1/2} \times 3$
